# Evolutionary dynamics of early and late mutational signatures in metastatic cancer

**DOI:** 10.3389/fbinf.2026.1735360

**Published:** 2026-03-05

**Authors:** Anastasia Yankovskiy, Sudhir Kumar, Sayaka Miura

**Affiliations:** 1 Department of Biology, Temple University, Philadelphia, PA, United States; 2 Institute of Genomic and Evolutionary Medicine, Temple University, Philadelphia, PA, United States; 3 Department of Biology, University of Mississippi, Oxford, MS, United States

**Keywords:** metastasis, mutational process, mutational signature, somatic mutation, tumor evolution

## Abstract

Cancer genomes accumulate somatic mutations over time, influenced by both intrinsic and extrinsic mutational processes. In metastatic cancer, disseminated tumor cells may acquire additional mutations at metastatic sites, shaped by extrinsic factors distinct from those at the primary tumor. As a result, cancer genomes at metastatic sites may bear mutational signatures originating from both primary and metastatic environments. However, the patterns and relative contributions of mutational signatures specific to metastatic sites remain poorly understood. To investigate this, we analyzed mutational signatures from seven metastatic cancer patients. We observed distinct mutational patterns between early and late mutation profiles within individual patients, where the early and late categories were based on their relative timing during tumor evolution. Early mutations were often dominated by a single mutational signature that accounted for more than half of the total signature burden. These dominant signatures tended to be shared among tumors of the same cancer type, suggesting that early mutations in metastatic cancers may be shaped by a single, highly active mutational process at the primary tumor site. In contrast, late mutations were often more poorly decomposed into distinct mutational signatures, reflecting more complex and diverse compositions. Overall, early mutations tended to preserve clearer signals of their origin.

## 1 Introduction

In cancer, mutations arise in individual cells and are passed down to their progeny, leading to genetically diverse populations of cells within a single patient, which leads to intratumor heterogeneity (Morris et al., 2016; Venkatesan and Swanton, 2016; Davis et al., 2017; Rosenthal et al., 2017; Dentro et al., 2021). As the disease progresses, cells with distinct mutations may acquire invasive traits, enabling metastasis to distant tissues where additional mutations can accumulate (Turajlic and Swanton, 2016; Tang et al., 2020; Patel et al., 2021; Chroni et al., 2022; Dymerska and Marusiak, 2024).

Mutational signatures are distinctive patterns of single-base substitutions found in specific trinucleotide contexts (Alexandrov et al., 2013; Alexandrov et al., 2020). They are influenced by biological and environmental processes and are described using a 96-channel trinucleotide framework (Alexandrov et al., 2013; Phillips, 2018; Alexandrov et al., 2020; Brady et al., 2022; Boysen et al., 2025). For example, SBS1 is associated with the age-related deamination of 5-methylcytosine, resulting in the substitution of cytosine with thymine (A[C>T]G). This mutation occurs at CpG sites, which are also known to exhibit approximately tenfold higher mutability, as observed in interspecies comparisons (Subramanian and Kumar, 2003). Other examples are SBS4 to tobacco carcinogen exposure and SBS13 to APOBEC cytidine deaminase activity (Alexandrov et al., 2013; Alexandrov et al., 2020). To determine the contribution of different signatures in a tumor, the observed mutational profile is computationally decomposed into known reference signatures, such as those curated in the COSMIC database (Tate et al., 2019; Sondka et al., 2024). Already, various computational approaches exist for such deconvolution (Rosenthal et al., 2016; Blokzijl et al., 2018; Huang et al., 2018; Li et al., 2020).

**FIGURE 1:**
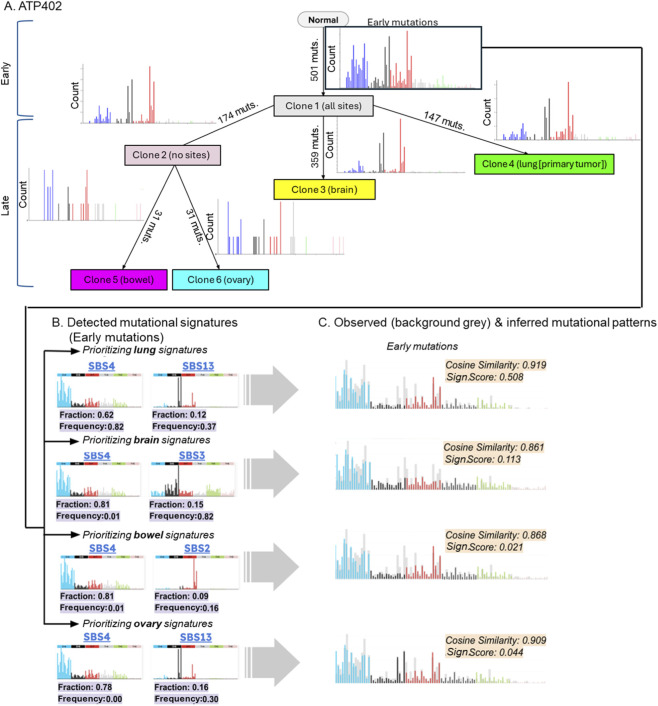
Mutational pattern of Patient ATP402. **(A)** Clone phylogeny and branch-specific mutation patterns of Patient ATP402. Clone phylogeny was inferred using observed read counts, and mutational patterns were mapped along branches. GenoPath pipeline (Tobin et al., 2025) was used, which implemented CloneFinder (Miura et al., 2018) for the inference of clone phylogeny. The number of mutations at each branch is shown along a branch. Tumor sites that had each clone are shown within a parenthesis next to a clone ID. **(B)** Detected mutational signatures for early mutations. The detection of signatures was optimized for those expected at each tumor site. The top two signatures with the largest estimated fractions are shown. Estimated fraction and population frequencies reported among the patients with the same cancer type are shown at the bottom. **(C)** Estimated mutational patterns based on the detected mutational signatures (early mutation). Observed mutational patterns are shown with gray on the background. Cosine similarity and Signature (Sign.) Score are shown for each early mutational profile.

Changes in mutational processes during tumor evolution have been reported (de Bruin et al., 2014; Barry et al., 2018; Ashley et al., 2019). Because metastatic lesions evolve in distinct microenvironments from the primary tumor, they may experience different mutational pressures that reshape the mutational landscape. However, how mutational signatures shift during metastatic progression remains largely unexplored. To address this question, we analyzed somatic mutations from seven metastatic cancer patients, each with multiple tumor samples from both primary and metastatic sites. Figure 1A shows an inferred phylogeny of cancer cell populations (clones) within a patient, where Clone 3, Clone 5, and Clone 6 are found only within metastatic tumor sites (brain, bowel, and ovary, respectively). Thus, unique mutations within each of these clones should have originated in their respective metastatic environments. However, the number of such site-specific mutations is often small (∼30 per site), limiting the ability to characterize mutational patterns. Therefore, we classified mutations shared across all tumor samples as “early” and pooled all non-early mutations as “late.” These late mutations are more likely than early mutations to include variants arising in metastatic lesions. Although late mutations may include some mutations that arise in the primary tumor site, they may predominantly reflect the mutagenic processes active after metastasis. We, therefore, compared mutational signatures between early and late mutations.

## 2 Methods

### 2.1 Annotation of early and late mutations

We analyzed data from seven patients, which came from a cohort of 40 metastatic cancer patients. Many patients were excluded from downstream analysis due to missing primary tumor samples or a low number of somatic mutations (<100 mutations in the early or late categories or lack of clear trinucleotide substitution patterns).

For the seven patients, we obtained single-nucleotide variants (SNVs) from the supplementary materials of a previous study, which performed whole-exome sequencing on these primary and metastatic tumor samples (Zhao et al., 2016). From this dataset, we extracted the observed read counts of both mutant and reference (non-mutant) bases at each SNV position for each tumor sample. For each sample, if the mutant base was detected at a given position, we recorded it in the sample’s SNV profile. Each SNV profile included the genomic position, the observed mutant base, and the surrounding trinucleotide context.

To annotate early and late mutations, we followed the approach described in a previous study (Gomez et al., 2018). Mutations shared by all samples from the same patient were annotated as early mutations, which are expected to be enriched for events that occurred prior to metastatic dissemination. In contrast, mutations not shared by all tumor samples were annotated as late mutations, which are more likely to include mutations that arose after metastasis.

### 2.2 Inference of relative fractions of mutational signatures

**FIGURE 2:**
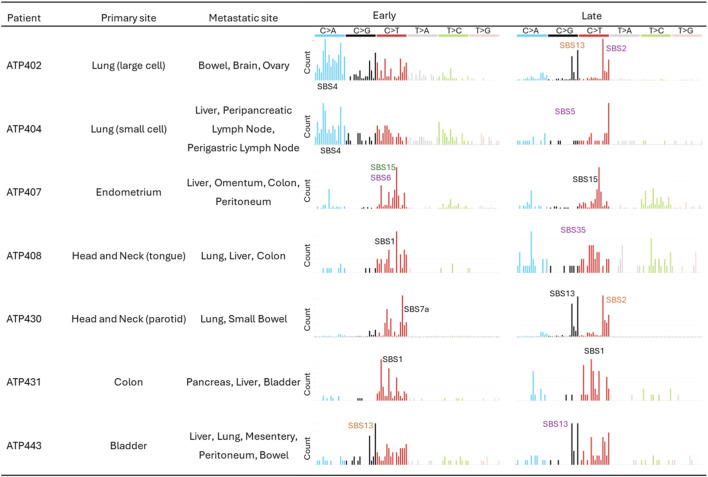
Early and late mutations of each patient. For each patient, primary and metastatic tumor sites are listed. When Signal software did not have the tumor site reported in the original study, we selected the closest tissue type. In this case, the tumor site reported in the original study was presented in parentheses. Mutational patterns are shown for early and late mutations. The characteristics of detected signatures with >50% fraction are noted with their signature IDs. Purple letters indicate that the signature was detected when the detection of signatures expected at a metastatic tumor site was optimized. Green and black letters are for primary tumor sites and both sites, respectively. When the characteristics of detected signatures with <50% fraction were clear, they were noted with orange.

To estimate the relative contributions of known mutational signatures, we applied the Signal software (Degasperi et al., 2020) separately to the early and late mutation profiles of each patient. The Signal requires a specified cancer type to guide the selection of relevant signatures. To evaluate how the choice of tumor site (primary vs. metastatic) influenced the identified signature profiles, we ran multiple separate analyses for each mutation profile: one using the primary tumor site and the other using each metastatic tumor site as the cancer type input. Tumor site information was obtained from the original study (Zhao et al., 2016). When a reported tumor site was not included among Signal’s supported cancer types, we selected the closest anatomically or biologically relevant tissue type as a proxy. The tumor sites used for Signal input, along with those reported in the original study, are shown in Figure 2 and Supplementary Table S1.

### 2.3 Assessment of identified mutational signatures

To evaluate how well the inferred signatures explain the observed mutational pattern, Signal calculates the cosine similarity (*CS*_*m*_) between the observed and estimated 96-channel trinucleotide profiles for each sample, *m*. The estimated trinucleotide profile is generated based on the combination of detected mutational signatures, their relative contributions, and the observed total mutation count within the sample.

While Signal prioritizes the detection of mutational signatures commonly observed for a given cancer type, unexpected signatures may be identified when the expected ones do not adequately fit the observed mutation pattern (Figure 1B). To penalize the inclusion of mutational signatures that are unexpected for a given cancer type, we incorporated a frequency-based weighting scheme (Figure 1C). Specifically, we weighted each detected signature, *i*, by its reported frequency among patients (*f*_*i*_) in the corresponding cancer type. We obtained *f*_*i*_ in Signal’s reference database (Supplementary Table S2). We defined the Signature Score (*SS*_*m*_) for each mutation profile *m* as:

$$SS_{m}=\left[\Sigma_{i}\;\left(S_{i}\times f_{i}\right)\right]\times CS_{m},$$ (1)

where *S*_*i*_ is the estimated fraction of mutational signature, *i*. This Signature Score reflects both the goodness-of-fit between observed and reconstructed mutation profiles, and the biological plausibility of the inferred signature composition based on known cancer-type-specific frequency (prevalence).

To calculate standard errors (SE) for *SS*_*m*_, we considered the estimation error of reported signature frequencies among patients in the Signal database,

$$\text{SE}=CS_{m}\times \sqrt{\Sigma_{i}}\left[S_{i}^{2}\times f_{i}\left(1\;–\;f_{i}\right)/N)\right],$$ (2)

where *N* is the number of patients in the database used to estimate the frequency, *f*_*i*_. Each signature frequency in the population was assumed to be independent. Because the signature fraction (*S*_*i*_) and cosine similarity (*CS*_*m*_) were point estimates and their variances were unknown, we could not include these uncertainties in the SE calculation. As a result, the SE values were likely underestimated. Additionally, we used these SE and performed pairwise t-tests for each Signature Score comparison.

## 3 Results

### 3.1 Temporal and spatial differences in mutational signatures: focus on early mutations

To investigate temporal changes in mutational processes, we classified somatic mutations into early and late categories by comparing primary and metastatic tumor samples from the same patients (see Methods). Overall, we observed visually distinct mutational patterns between early and late mutations across multiple patients (Figure 2). For example, in Patient ATP402, who had a primary lung tumor with metastases in the brain, bowel, and ovary, late mutations were dominated by C>G (notably T[C>G]A and T[C>G]T) and C>T substitutions (T[C>T]A and T[C>T]T), while early mutations additionally had many C>A substitutions. We therefore hypothesized that the contribution of metastatic tumor sites to late mutations may underlie the observed shift in mutational profiles, reducing the dominance of signatures from primary tumor sites.

**FIGURE 3:**
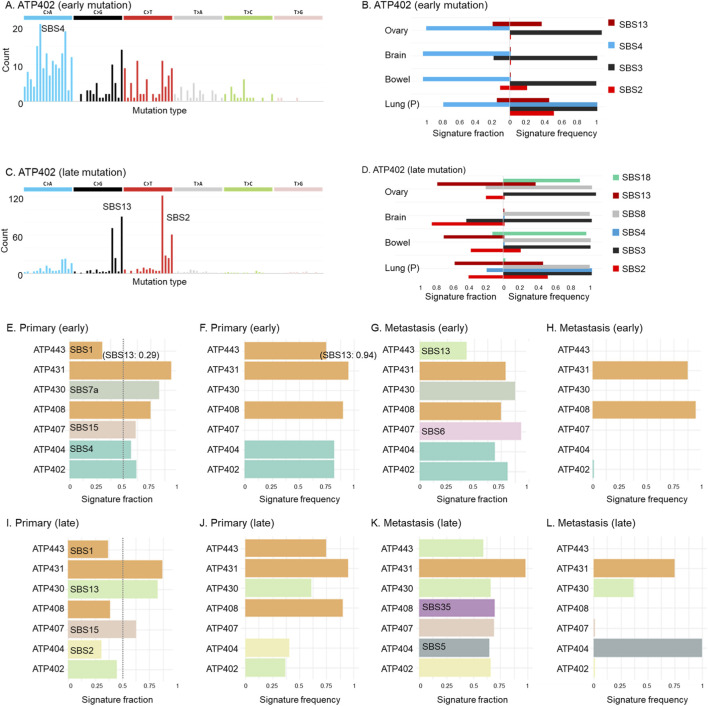
Detected mutational signatures and population frequencies (prevalences) found within given tumor sites. **(A–D)** Observed mutational patterns and detected mutational signatures of Patient ATP402. Early mutations **(A,B)** and late mutations are shown **(C,D)**. The high frequency of C>A mutations is the characteristic of SBS4 for the early mutation **(A)**, and the characteristics of SBS13 and SBS2 are indicated for the late mutations **(C)**. The fractions of detected mutational signatures and their frequencies among patients are shown **(B,D)**. The detections of mutational signatures were optimized for the signatures expected at each tumor site. **(E–L)** Dominant mutational signatures of early **(E–H)** and late times **(I–L)** for all the patients. The largest estimated fractions of signatures are shown **(E,G,I,K)**. The detections of signatures at the primary **(E,I)** and metastatic **(G,K)** tumor sites were optimized. A dotted line indicates the fraction of 0.5 **(E,I)**. The reported frequency of patients in the Signal database is shown **(F,H,J,L)**. When the detection of signatures at a metastatic tumor site was optimized, the tumor site with the largest dominant signature fraction was shown **(G,H,K,L)**. While the dominant signature was SBS1 for patient ATP443, SBS13 was also detected with almost the same fraction as SBS1 (0.29) **(E)**. Therefore, we presented SBS13 next to SBS1 in a parenthesis. SBS13 also had a high frequency at the primary tumor site (0.94), and this information was shown in a parenthesis **(F)**.

To test this, we first used the Signal software to infer the relative fractions of known mutational signatures in the early mutation profiles for each patient. In the case of Patient ATP402, the early mutations showed a strong enrichment for SBS4, which accounted for 62% of the estimated signature composition (Figures 3A,B). SBS4 is commonly associated with exposure to tobacco-related carcinogens and was detected in 81% of lung cancer patients in the Signal reference database. This SBS4 signature is characterized by frequent C>A substitutions, which were also prominent in ATP402’s early mutations, suggesting that these mutations likely originated in the lung, which was the primary tumor site of this patient.

Next, we reran the analysis using metastatic tumor sites as the input cancer type. For Patient ATP402, SBS4 remained the dominant signature (Figure 3B). Because none of the metastatic sites are typically associated with SBS4, this result further supports the conclusion that early mutations in this patient were primarily acquired in the primary lung tumor, prior to metastatic spread.

We found that this pattern of the early mutations could extend beyond Patient ATP402. In nearly all cases, one dominant signature accounted for more than half of the mutational burden, when the detections of the mutational signatures at the primary tumor site were optimized (Figure 3E; Supplementary Table S1). The only exception was Patient ATP443, where the dominant signature SBS1 accounted for less than 50% of the early mutations as SBS13 additionally had clear contribution (Figure 2).

For four out of six patients, these major signatures were also recurrent within their respective cancer types, appearing in over 75% of patients in the Signal reference cohort (Figure 3F; Supplementary Table S2). The exceptions were Patients ATP430 and ATP407. However, their dominant signatures (each >50%) did not well align with the observed trinucleotide mutation patterns (Figure 2). This observation implied a mixture of multiple weaker signatures or the presence of atypical, rare signatures, leading to incorrect decomposition into a single dominant signature.

We lastly found that the same major signatures persisted when the analysis was repeated by optimizing the detection of signatures expected at the metastatic tumor sites (six out of the seven patients) (Figure 3G). We further found that these signatures were not associated with the metastatic tissues except for the SBS1, which is commonly found in various cancer types (Figure 3H).

### 3.2 Late mutations: diverse and mixed signatures

We further analyzed late mutations. For example, in Patient ATP402, we found that the early mutations were dominated by SBS4, whereas in the late mutations, SBS13 became the most prominent signature, followed closely by SBS2 (Figures 3C,D). Both SBS13 and SBS2 are APOBEC-associated signatures, and they are expected to be observed at the primary tumor site (lung). We further found that SBS13 was also expected in one of ATP402’s metastatic tumor sites (ovary), and SBS2 was in another site (bowel) (Figure 3D). Therefore, whether these late mutations originated in the primary tumor or at metastatic sites could not be determined from this result.

Similarly, for approximately half of the patients (four, including ATP402), the dominant mutational signature in late mutations accounted for less than 50% of the total mutational burden when detection was optimized using signatures expected at the primary tumor site (Figure 3I), while these signatures were generally expected to be observed at the primary tumor sites (Figure 3J).

We next optimized the detection of signatures that were characteristic at the metastatic tumor sites. Interestingly, we detected high-frequency dominant signatures (>50%) in all patients, which was different from when primary tumor sites were optimized for the signature detection (Figure 3K). However, these signatures were not expected for the metastatic sites in question or were expected at both primary and metastatic tumor sites, except for ATP404 (Figure 3L). In addition, these signatures often did not well align with the observed mutational patterns, suggesting that these dominant signatures were likely spuriously detected due to the difficulty in correctly decomposing the mixture of multiple signatures (Figure 2).

### 3.3 Contribution of mutational processes from primary and metastatic tumor sites

Finally, we quantitatively evaluated how well the detected mutational signatures explained the observed mutation profiles. To do this, we compared the observed mutation patterns to those estimated from detected signatures using cosine similarity and our newly proposed Signature Score, which accounts for the biological relevance of detected signatures (see Methods).

**FIGURE 4:**
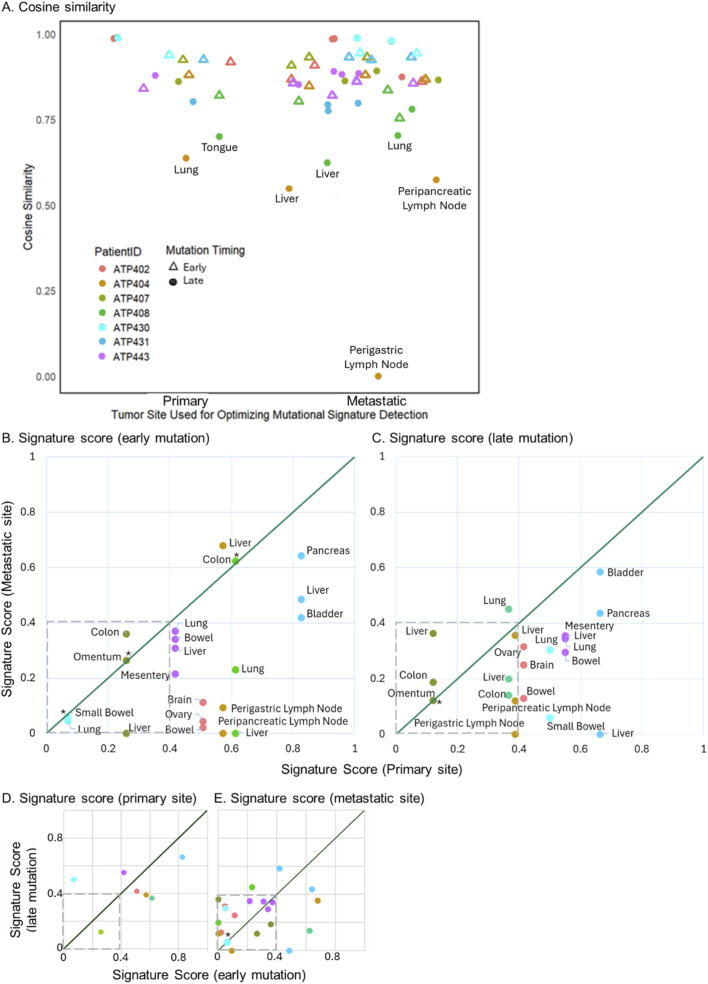
Cosine similarity and Signature Score. **(A)** Cosine similarity. Cosine similarity was calculated for each tumor sample by optimizing the detection of mutational signatures expected at primary and metastatic sites. A triangle and circle represent early and late mutations, respectively. A t-test was performed, and the difference in cosine similarity between primary and metastatic sites was not statistically significant for early (*p* = 0.55) and late mutations (*p* = 0.49). Similarly, the difference in cosine similarity between early and late mutations was not statistically significant for primary (*p* = 0.33) and metastatic sites (*p* = 0.08). **(B–E)** Signature Score. For early **(B)** and late **(C)** mutations, Signature Scores between primary and metastatic tumors are compared. For primary **(D)** and metastatic **(E)** tumor sites optimization, Signature Scores between early and late mutations were compared. Standard errors of Signature Scores (Equation 2) are shown in Supplementary Table S3. P-values with >0.05 are shown with *. The actual values are listed in Supplementary Table S4. Low Signature Scores were indicated within dashed boxes.

We first analyzed cosine similarity, which measures how closely the reconstructed mutational profile matches the observed one. Across most patients, cosine similarity was similar between early and late mutations, regardless of whether we optimized signature detection for the primary or metastatic tumor sites (Figure 4A). Nevertheless, a few patients (outliers) showed noticeably lower cosine similarity for late mutations. These outliers probably imply a complex mixture of different mutational signatures or atypical signatures because signature decomposition is difficult for such cases, i.e., inaccurate signature decomposition potentially lowered the cosine similarity.

However, it is important to note that cosine similarity can be high even when the detected signatures are unexpected for a given tumor site. For example, in Patient ATP402, the early mutations were characterized with excess of C to A mutations, which is the characteristics of SBS4 signature, and SBS4 is expected to be observed within only the primary tumor site (lung) (Figure 1A). However, cosine similarity was high (∼92%) regardless whether we used primary or metastatic tumor site–specific signatures for optimization (Figure 1C). This was because the dominant signature, SBS4, was detected at high levels in both cases, even though SBS4 is only expected at the primary site (Figure 1B).

To address this limitation, we applied our Signature Score, which explicitly penalizes the detection of signatures that are not expected at a given tumor site (Equation 1). In the case of ATP402, the Signature Score was substantially higher for the primary tumor site than for the metastatic sites, correctly indicating that the mutational processes were more consistent with the primary site (Figure 1C).

We, therefore, extended this analysis across all patients. For early mutations, Signature Scores were generally higher when optimized for the primary tumor sites than metastatic sites, supporting the interpretation that early mutations predominantly arose within the primary site (Figure 4B; Supplementary Table S3). Only two metastatic tumors (liver from Patient ATP404 and colon from Patient ATP408) had a slightly higher or similar signature score than the primary site. For patient ATP404, the dominant signature detected was SBS4, which is expected in both the lung primary tumor and liver metastasis. Accordingly, both sites showed similarly high scores (0.57 in the primary and 0.68 in the liver). While the liver showed a statistically significant higher score, the absolute difference (0.1) appears small and is unlikely to be biologically meaningful. Similarly, for the other patient, ATP408, both the primary tumor and the colon metastasis were dominated by SBS2 and SBS1, which are also expected in both tissues, resulting in nearly identical scores (0.61 and 0.62). Thus, whether early mutations arose within the primary sites was unclear for these two cases.

We also observed that two patients (ATP430 and ATP407) had relatively low Signature Scores for both primary and metastatic sites (<0.4). This indicates that neither profile was well explained by the reference signatures. This likely reflects a mixture of multiple weaker signatures, or atypical, rare signatures may have been incorrectly decomposed, as discussed earlier.

For late mutations, Signature Scores were still often higher for the primary tumor site compared to each individual metastatic site (Figure 4C). A possible explanation is that a substantial fraction of late mutations were still accumulated while the tumor cells resided in the primary site, before dissemination. As a result, their mutational profiles were still better explained by signatures associated with the primary tumor site.

At the same time, Signature Scores for late mutations were generally lower than for early mutations when detection of primary signatures was prioritized, suggesting that late mutations were shaped by a broader mix of mutational processes (Figure 4D). On the other hand, most metastatic sites had low scores for both early and late mutations (Figure 4E). This indicates that many metastatic sites do not account for the majority of late mutations.

## 4 Discussion

Our analysis suggested a difference in the mutational landscapes of early and late mutations. Early mutations within a patient often arose under the influence of a single dominant mutational process in the primary tumor site. In contrast, late mutations may be shaped by multiple mutational processes, leading to a more heterogeneous mutational landscape.

We initially aimed to compare mutational signatures between early and late mutations using the ideal framework of mapping signatures directly onto the branches of clonal phylogenies (Miura et al., 2018; Miura et al., 2022; Kumar et al., 2020; Tobin et al., 2025). This approach would enable precise reconstruction of tumor evolutionary history, including direct tracking of tumor cell migration and pinpointing the tumor site in which each mutation originated. Such branch-level resolution is critical for understanding how mutational processes shift over time and across anatomical sites. However, in our dataset, the number of mutations assigned to each branch was often small (Figure 1A). Because accurate signature assignment requires a substantial number of mutations (Miura et al., 2022), we were not able to perform mutational signature deconvolution at each branch of a clone phylogeny in this study.

In addition, the small cohort size limits the generalizability of the observed patterns in this study. Moving forward, we plan to validate and refine these findings using bigger cohorts with whole-genome sequencing (WGS), which provide orders of magnitude more mutations per branch, enabling branch-level evolutionary mapping of mutational processes.

It is also important to note that the classification of mutations as early or late is sensitive to sampling completeness, particularly to the number of metastatic sites analyzed per patient. If some metastatic lesions are missing, mutations that are truly late may appear shared across all sampled tumors and thus be misclassified as early. Nevertheless, in our data, the primary tumor site often showed higher signature scores for early mutations than for late mutations, suggesting that incomplete sampling of metastatic sites is unlikely to change our main conclusion.

Clinically, accurately identifying the primary tumor site is essential for diagnosis, prognosis, and treatment planning (Varadhachary, 2007; Tomuleasa et al., 2017). In some patients, however, the primary site cannot be determined using standard diagnostic tools such as immunohistochemistry or targeted biopsies, particularly when the tumor is very small, has regressed, or has been eliminated by the immune system (Pavlidis and Pentheroudakis, 2012; Tomuleasa et al., 2017). Such cases are classified as cancer of unknown primary (CUP), which accounts for approximately 2%–5% of all cancers and is associated with poorer patient outcomes due to inaccuracies in treatment selection (Varadhachary, 2007). To address this challenge, computational approaches have been developed to predict tumor origin, including methods that analyze mutational patterns from tumor genomes (Dietlein and Eschner, 2014; Chen et al., 2015). These approaches, however, use the full set of mutations without considering the timing of their acquisition.

In our analysis, we repeatedly prioritized the detection of mutational signatures expected for each tumor site and then evaluated which prioritization best explained the observed mutational patterns. We found that prioritization corresponding to the primary tumor site often provided a better fit than that corresponding to metastatic sites. Also, early mutations preserved a more distinct “molecular fingerprint” of the tissue of origin. Incorporating early/late mutation stratification into tumor-origin prediction algorithms could therefore improve predictive accuracy for CUP cases.

However, some mutational signatures are commonly observed in more than one tumor site, making the distinction difficult. For example, Patient ATP408 had a similar Signature Score for early mutations between the primary (tongue) and one of the metastatic tumors (colon), so we cannot tell primary tumor site from mutational signatures of early mutations (Figure 4B). Nevertheless, since the other two metastatic tumor sites had much lower Signature Scores (lung and liver), we can at least eliminate a few candidate primary tumor sites. In the future, we aim to test this strategy and evaluate its potential for clinical application.

## 5 Conclusion

Our findings highlight the value of separating early from late mutations for metastatic cancer data analysis. Early mutations retain clearer signals of their origin, offering a promising avenue for improving primary tumor site prediction.

## Data Availability

The original contributions presented in the study are included in the article/Supplementary Material, further inquiries can be directed to the corresponding author.
